# Exogenous Nitric Oxide (NO) Interferes with Lead (Pb)-Induced Toxicity by Detoxifying Reactive Oxygen Species in Hydroponically Grown Wheat (*Triticum aestivum*) Roots

**DOI:** 10.1371/journal.pone.0138713

**Published:** 2015-09-24

**Authors:** Gurpreet Kaur, Harminder Pal Singh, Daizy R. Batish, Priyanka Mahajan, Ravinder Kumar Kohli, Valbha Rishi

**Affiliations:** 1 Department of Environment Studies, Panjab University, Chandigarh, 160 014, India; 2 Department of Botany, Panjab University, Chandigarh, 160 014, India; 3 Chandigarh College of Engineering and Technology, Chandigarh-160019, India; Chinese Academy of Sciences, CHINA

## Abstract

Nitric Oxide (NO) is a bioactive signaling molecule that mediates a variety of biotic and abiotic stresses. The present study investigated the role of NO (as SNP [sodium nitroprusside]) in ameliorating lead (Pb)-toxicity in *Triticum aestivum* (wheat) roots. Pb (50 and 250 μM) alone and in combination with SNP (100 μM) was given to hydroponically grown wheat roots for a period of 0–8 h. NO supplementation reduced the accumulation of oxidative stress markers (malondialdehyde, conjugated dienes, hydroxyl ions and superoxide anion) and decreased the antioxidant enzyme activity in wheat roots particularly up to 6 h, thereby suggesting its role as an antioxidant. NO ameliorated Pb-induced membrane damage in wheat roots as evidenced by decreased ion-leakage and *in situ* histochemical localization. Pb-exposure significantly decreased *in vivo* NO level. The study concludes that exogenous NO partially ameliorates Pb-toxicity, but could not restore the plant growth on prolonged Pb-exposure.

## Introduction

Various anthropogenic activities, such as disposal of sewage sludge and electronic waste, mining and smelting have resulted in augmentation of heavy metals in the natural environment [1]. Upon accumulation in the soil, heavy metals may disturb soil biology and ecology; thereby inhibiting plant growth, affecting crop produce and even causing plant death [1, 2]. The phytotoxic effects of heavy metals have been attributed to pro-oxidative effects of metal ions, that disrupt various physiological and biochemical processes [2]. Lead (Pb) has been reported to induce reactive oxygen species (ROS) generation and alter enzymatic machinery in lupin roots, pea root cells, rice plants, maize roots, and aquatic plants such as *Wolffia arrhiza* and *Elodea canadensis* [3].

Nitric Oxide (NO), a free radical bioactive molecule, plays a significant role in plant signaling networks [4, 5]. In plants, NO is involved in many physiological and developmental processes such as breaking of seed dormancy and germination, flowering, root organogenesis, plant development, senescence, interaction with plant hormones, and defense against biotic and abiotic stresses, maintaining redox balance and root iron homeostasis [6, 7, 8].

Following metal exposure, NO supplementation is reported to protect against oxidative stress, ion leakage, DNA fragmentation, and cell death [9]. NO is a highly reactive molecule and allows extremely rapid reactions with other free radicals to terminate the chain propagated reactions [10].

NO interacts with ROS and other antioxidants and prooxidants [11]. The cross-talk between NO and ROS signaling pathways regulates the hypersensitive response, programmed cell death and senescence in plants [6, 12, 13, 14]. During senescence, ROS plays a positive role, whereas NO can either incite or hinder the senescence, and the effect is dependent on concentration and sub-cellular location. NO may ameliorate the ROS-induced toxicity and can act as a delaying factor for leaf senescence in plants [15, 16]. Early leaf senescence was reported in the NO-deficient mutant *nos1/noa1* [16], and similarly, senescence like phenotype was displayed by *Arabidopsis* expressing NOD, an NO degrading dioxygenase [15]. The cross-talk between NO and phytohormones regulates early plant developmental processes such as seed dormancy and germination, hypocotyl elongation and root development [17].

Exogenous NO provides protection to plants against water [18], photo-oxidative [19], and heavy-metal (such as Al, As, Cu, Cd) [9] stress. NO supplementation has been found to reduce Pb uptake in *Arabidopsis thaliana*, thereby reducing toxicity symptoms [20]. However, there is a paucity of information regarding the role of NO in alleviating Pb-induced toxicity. NO also influences gene expression in response to oxidative stress in *Zea mays* leaves [21]. Previously, we found that SNP (an NO donor) supplementation to Cd significantly reduced the Cd-induced lipid peroxidation, H_2_O_2_ accumulation and enhanced electrolyte leakage in wheat after 24 h of treatment, thus, indicating ROS scavenging activity of NO [22]. With this background in mind, we hypothesized that NO may ameliorate Pb-induced toxic effects in wheat. We, therefore, investigated the antioxidant role of NO in regulating Pb-induced oxidative damage in hydroponically grown wheat roots. In addition, the effect of exogenous NO on ROS (lipid peroxidation, hydrogen peroxide, conjugated dienes, superoxide ion, and hydroxyl ion content) generation, membrane disintegration, ROS metabolism; changes in protein content and alterations in SOD and CAT activity at molecular level were explored during 0–8 h of Pb-exposure.

## Material and Methods

### Materials

Healthy seeds of wheat (*Triticum aestivum*. var. PBW 502) were purchased locally from the market. These were surface sterilized with sodium hypochlorite (0.1%, *w/v*) and washed under running tap water followed by rinsing in distilled water. Pb was supplied in the form of lead nitrate (MW = 331.21; purity = 99%; Merck Ltd., Mumbai, India). All the reagents and chemicals used for biochemical analysis were of technical grade and procured from Sisco Research Laboratory Pvt. Ltd., India; Sigma Co., St. Louis, USA; Merck Ltd., India; Acros, Belgium; and Loba-Chemie Pvt. Ltd., India.

### Experimental Set-Up: Hydroponic Growth Conditions

Wheat seeds (imbibed in distilled water for 6 h at 25°C) were germinated on wet filter paper in enamel trays (32 cm × 23 cm × 7 cm) lined with a moist cotton wad. After 24 h, germinating seeds were transferred to nylon mesh floating on distilled water in glass beakers (500 ml capacity) in a growth chamber set at day /night temperature of 20/10 (±2) °C, a relative humidity of 75±3%, and a photoperiod of 12 h at a photosynthetic photon flux density (PPFD) of ~240 μmol photons m^−2^ s^−1^. After 48 h, when the average root length was ~ 2.5 cm long, different treatments were given.

### Treatments and Plant Harvest

A preliminary experiment with SNP at 0, 10, 50, 100, 200 and 400 μM was performed to determine the point where SNP showed the most significant effect. NO donor SNP at 100 μM had the promotory effect on the root length of wheat seedlings whereas a decline in root length was observed at ≥200 μM. There were a total of 6 treatments, including control, with 5 replicates in each. These include: (i) Distilled water alone (control; without any additional nutrients/minerals); (ii) distilled water + SNP (100 μM); (iii) 50 μM lead nitrate (hereafter, referred as Pb); (iv) 50 μM Pb+100 μM SNP; (v) 250 μM Pb; and (vi) 250 μM Pb+100 μM SNP. The experiment was carried out in a completely randomized block design manner. After 0, 2, 4, 6 and 8 h of treatment, wheat seedlings were harvested; their radicle and coleoptile lengths were measured.

Since the effect of Pb-toxicity was more pronounced on roots, these were excised, washed with 10 mM CaCl_2_, and stored at –20°C. These were used for further biochemical studies, histochemical analyses and assessment of oxidative damage.

### ROS Generation

#### Hydrogen peroxide (H_2_O_2_) Content

One hundred mg of root tissue was extracted with 5 ml of trichloroacetic acid (TCA; 0.1%, *w/v*) in an ice bath and centrifuged at 12,000×*g* for 15 min [22]. An aliquot (0.5 ml) of the supernatant was added to 0.5 ml of PO_4_^3−^ buffer (pH = 7.0) and 1 ml of 1M KI. The absorbance of the mixture was recorded at 390 nm. H_2_O_2_ content was determined using the molar extinction coefficient (ε) of 0.28 μM^−1^cm^−1^ and the amount was expressed as nM g^−1^ FW (fresh weight).

#### Superoxide anion (O_2_^●−^) content

Root tissue (100 mg) was homogenized in 10 ml of 0.1 M PO_4_^3−^ buffer (pH = 7.0) in a pre-chilled pestle mortar. The homogenate was centrifuged at 15,000×*g* for 20 min at 4°C. To 0.2 ml of extract, 1.8 ml of 1 mM adrenaline prepared in 75 mM PO_4_^3−^ buffer (pH 7.4) was added. Absorbance of the mixture was read at 480 nm immediately after the addition of the enzyme extract and then again after 5 min. The amount of O_2_^●−^ was calculated using ε = 4020 M^–1^cm^–1^ and expressed as μM g^–1^ FW [23].

#### Hydroxyl ion (^●^HO) content

For estimation of extracellular ^**●**^HO, 10 excised root tips of equal length weighing approximately 50 mg were incubated in 1 ml of 10 mM PO_4_^3−^ buffer (pH = 7.4) containing 15 mM 2-deoxyderibose, at 37°C for 2 h [24]. Following incubation, an aliquot of 0.7 ml from the above mixture was added to the reaction mixture containing 3 ml of 0.5% (*w/v*) thiobarbituric acid (TBA, 1% stock solution made in 5mM NaOH) and 1 ml of glacial acetic acid. The contents of the reaction mixture were heated in a water bath for 30 min at 100°C, and then cooled down to 4°C for 10 min. The absorbance of the reaction mixture was measured at 532 nm. The content of ^**●**^HO was calculated using ε = 155 mM^−1^cm^−1^ and expressed as nM g^−1^ FW.

### Lipid Peroxidation

Lipid peroxidation was determined by measuring the amount of MDA (malondialdehyde), a thiobarbituric acid reactive species (TBARS) [22]. One hundred mg of roots were homogenized in 5 ml of 0.1% TCA (*w/v*) and centrifuged at 10,000×*g* for 10 min. One ml of supernatant was mixed with 4 ml of 0.5% thiobarbituric acid (TBA) in 20% TCA. The mixture was heated at 95°C for 30 min, cooled over ice, and centrifuged at 10,000×*g* for 10 min. The absorbance of the supernatant was recorded at 532 nm and corrected for non-specific turbidity by subtracting the non-specific absorbance at 600 nm. MDA content was calculated using ε = 155 mM^−1^cm^−1^ and expressed as nM g^−1^ FW.

### Conjugated Diene (CD) Quantification

One hundred milligram of root tissue was homogenized in 5 ml of 95% (*v/v*) ethyl alcohol. The mixture was centrifuged at 10,000×*g* for 10 min, and the absorbance was read at 234 nm. The content of conjugated dienes was determined by using ε = 26.5 mM^−1^ cm^−1^ and expressed as μM g^−1^ FW [25].

### In Situ Nitric Oxide Detection and Measurement of Nitrite (NO_2_^−^)

Nitric oxide was detected histochemically using NO-sensitive fluorescent probe 4,5-diaminofiuorescien diacetate (DAF-2DA) and epifluorescence microscopy [26]. Roots were incubated in 5 μM DAF-2DA in 20 mM HEPES/NaOH buffer (pH = 7.5) for 2 h at 25°C in the dark. It was followed by washing with 20 mM HEPES/NaOH buffer (pH = 7.5) thrice, for ten min each. NO was detected as green fluorescence of DAF-2DA using epifluorescence (excitation 480–485 nm; emission 515–535 nm) under bright-field microscope (DMLS, Leica Microsystems GmbH, Wetzlar, Germany) fitted with a digital microscope camera (ProgRes®, Jenoptik, Germany).

In addition, NO generation was also quantified by determination of nitrite (NO_2_^−^) concentration in vivo using Griess reagent. Samples (0.2 ml) were incubated with 1.8 ml of 100 mM PO_4_^3−^buffer (pH 7.0) and 0.2 ml of Griess reagent (1% sulfanilamide and 0.1% N-1-napthylethylenediamine dihydrochloride in 5% phosphoric acid solution) at room temperature for 10 min [27]. Absorbance of the reaction mixture was read at 540 nm and concentration of NO was determined from a calibration curve prepared using sodium nitrite as standard.

### Root Membrane Integrity

Root membrane integrity was studied in terms of relative electrolyte leakage (REL) from the roots [28]. Roots (200 mg) were incubated in distilled water at 25°C for 2 h in test tubes and initial conductivity (E_1_) of the bathing medium was measured. The tubes containing the root material were boiled for 30 min to release all the electrolytes. These were cooled to 25°C, and the conductivity (E_2_) was measured again. The electrolyte leakage was calculated as: REL (%) = (E_1_/ E_2_) × 100

### In Situ ROS Detection

In situ ROS generation was determined histochemically in terms of plasma membrane integrity (using Evans Blue solution, 0.025%, *w/v*) and O_2_^●–^ generation (using nitroblue tetrazolium; NBT, 0.05%, *w/v*) as per Singh *et al*. [29].

### ROS Metabolism

ROS metabolism was determined in terms of the activities of ROS scavenging enzymes, namely, superoxide dismutases (SOD), catalases (CAT), ascorbate peroxidases (APX), guaiacol peroxidases (GPX), and glutathione reductases (GR). Frozen root tissue (100 mg) was homogenized in 10 ml of 0.1 M PO_4_^3−^ buffer (pH = 7.0) in a pre-chilled pestle mortar. The homogenate was passed through three layers of cheese cloth followed by centrifugation at 15,000×*g* at 4°C (rotor temperature) for 30 min. The supernatant was stored at –20°C until used for the assay of enzyme activities. The protein content in the homogenates was determined according to the method of Bradford [30], using bovine serum albumin as the standard.SOD was assayed by measuring its capacity to inhibit photochemical reduction of NBT [22]. A 50% photoreduction of NBT at 25°C was considered as one unit of enzyme activity. CAT activity was determined by monitoring the disappearance of H_2_O_2_ at 240 nm and calculated using ε = 39.4 mM^−1^ cm^−1^ [22]. APX activity was determined as the oxidation of ascorbic acid at 290 nm and calculated using ε = 2.8 mM^−1^cm^−1^ [3]. GPX activity was determined at 470 nm in terms of guaiacol oxidized and calculated using ε = 26.6 mM^−1^cm^−1^ [22]. GR was measured by following nicotinamide adenine dinucleotide phosphate (NADPH) oxidation at 340 nm and determined using ε = 6.224 mM^−1^ cm^−1^ [22]. The specific activities of CAT, APX, GPX, and GR were expressed as enzyme unit (EU) mg^−1^ protein, and one EU is the enzyme that catalyses 1.0 mM of H_2_O_2_, ascorbate, guaiacol, or NADPH per min at 25°C, respectively.

### Protein Extraction

Root tissues were collected from the control as well as Pb and Pb+SNP treated seedlings after 8 h of the treatment. Samples (500 mg) were macerated in a pre-chilled mortar and pestle by adding 1.5 ml of 50 mM potassium PO_4_^3–^ buffer (pH = 7.8), 50 mM EDTA, 2 mM PMSF and 10% (*w/v*) PVP to a fine slurry, followed by centrifugation at 15,000×*g* for 15 min at 4°C. Similar extraction procedure was followed for SOD and CAT detection. The supernatants were stored at –20°C in small aliquots for analysis of protein estimation [30].

### SDS-PAGE Electrophoresis

The detection of total proteins, SOD and CAT activity was done by sodium dodecyl sulfate–polyacrylamide gel electrophoresis (SDS-PAGE) according to Laemmli [31]. The supernatant containing 25 μg of protein was mixed with sample buffer (12.5 ml Tris–HCl, pH = 6.8; 2.5 ml glycerol; 10%, *w/v*, SDS; 1.25 ml *β*–mercaptoethanol, *v/v*; and 0.01% bromophenol blue) in ratio of 4:1 and heated for 10 min at 90°C, cooled on ice before loading on 12% polyacrylamide slab gels. Electrophoresis was carried out at 70 V for 4 h using a SCIE-PLAS TV100YK Cooled Twin-Plate Mini-Gel Electrophoresis Unit (SCIE-PLAS, UK).

Mid Range 3 protein markers (14–95 kDa; Sisco Research Laboratories, Mumbai, India) were run simultaneously in the electrophoresis gel. For total protein analyses, the gels were stained with Coomassie brilliant blue for 2 h and kept in destaining solution (Methanol: Glacial Acetic Acid: Distilled water; 4:1:5, *v/v*) for overnight [32]. SOD was detected by soaking the gel in NBT solution (0.6 g in 100 ml of distilled water) with constant shaking for 20 min in dark [33]. After washing the gel for 2–3 times with distilled water, it was stained with a solution containing 0.0105 g of riboflavin and 0.5 ml of TEMED dissolved in 100 ml of 100 mM PO_4_^3–^ buffer (pH = 7.0) for 20 min in dark. Thereafter, the gels were washed again with distilled water, and exposed to white light. To stop the reaction, the gels were transferred to 6% (*v/v*) acetic acid. For CAT activity, the gels were firstly soaked in 0.3% H_2_O_2_ for 15 min [34]. Further, they were soaked in a solution containing 1% potassium ferricyanide and 1% ferric chloride (equivalent to 2% in total) for another 15 min. It was followed by washing thrice with distilled water, and then fixed with 1% HCl. Finally, the gels were scanned and photographed using gel documentation system (GelVisionDC; Biotron Healthcare, India).

### Data Analyses

All the experiments were performed in a randomized block design with five beakers, each beaker serving as a replicate. Each biochemical estimation and enzymatic analysis involved five independent tissue samples to serve as replications. The data are presented as mean±standard error (SE) and analyzed by one-way ANOVA followed by the separation of treatment means from respective controls at *p*<0.01 and *p*<0.05 applying post hoc Dunnett’s test, Tukey’s test and two sample *t*-test.

## Results

### Effect of NO on Seedling Growth of Wheat

After 8 h of Pb exposure, the radicle length was reduced by ~31% and 54% (*p*<0.05) at 50 and 250 μM Pb, respectively. Plumule growth was less sensitive to the toxic effects of Pb, with ~17% (50 μM) and 45% (250 μM) reduction relative to the control (Table 1). However, on SNP supplementation, although a reduction in radicle and plumule length was observed, but it was significantly lesser than Pb treatment alone. The reduction in radicle length was ~26% and 47% when exposed to Pb treatments (50 and 250 μM, respectively) supplemented with SNP (Table 1).

**Table 1:** Effect of NO (as SNP; 100 μM) on the Pb-induced changes in radicle and coleoptile length (in cm) of wheat seedlings determined after 8 h exposure to Pb (50 and 250 μM, as lead nitrate).

Treatment (μM)	Radicle length (cm)		Coleoptile length (cm)	
	–SNP	+SNP	–SNP	+SNP
0 (Control)	6.30±0.05a	7.22±0.12a	6.36±0.10a	6.87±0.0.09a
50 μM Pb	4.34±0.07b	4.68±0.12b*	5.28±0.08b	5.72±0.12b**
250 μM Pb	2.90±0.14c	3.32±0.09c*	3.50±0.07c	4.01±0.08ce**

Data represented as mean±SE of five independent replicates; Different alphabets within a column represent significant difference among treatments at *p*<0.05 applying post hoc Tukey’s test.

* and ** represent significant difference between treatments (with and without SNP) at *p*<0.05 and at *p*<0.01, respectively, applying two-sample *t-*test

### Effect of NO on Pb-Induced Lipid Peroxidation and Conjugated Diene (CD) Content

**Fig 1:**
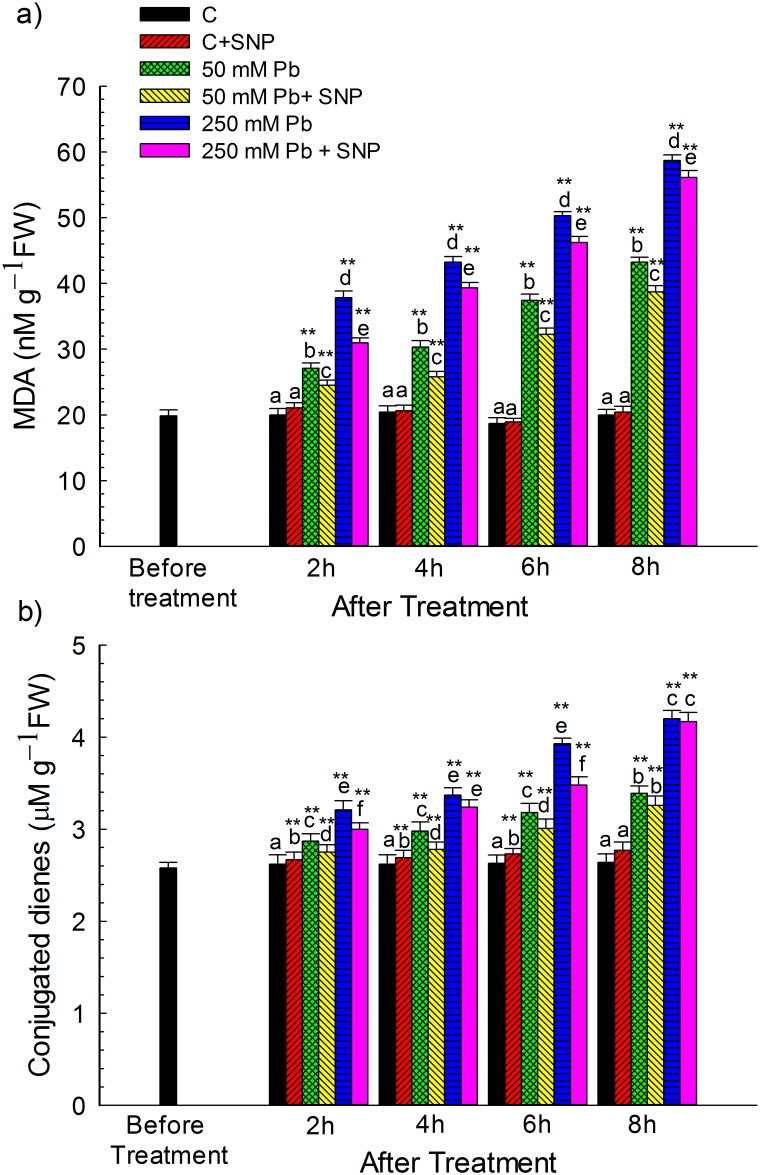
Effect of SNP (100 μM) on the Pb-induced changes in (a) lipid peroxidation (as MDA, nM g^–1^ FW) and (b) conjugated dienes (as CD, μM g^–1^ FW) of wheat seedlings determined before and after 2, 4, 6 and 8 h of exposure to Pb (50 and 250 μM, as lead nitrate). Data presented as mean±SE. * and ** represent significant difference of various treatments at 2, 4, 6 and 8 h stage from 0 h stage at *p*<0.05 and at *p*<0.01, respectively, applying post hoc Dunnett’s test. Different letters within a particular stage (2, 4, 6 or 8 h) represent significant difference at *p*<0.05 applying post hoc Tukey’s test.

MDA content was ~36% and 89% higher than control after 2 h of exposure to 50 and 250 μM Pb, respectively. It increased further and was 116% and 194% greater over the control after 8 h of exposure to 50 and 250 μM Pb, respectively (Fig 1a). Compared with Pb treatment alone, NO addition caused a significant decrease in MDA levels during 2–8 h of treatment. It reduced MDA levels in 50 μM Pb treatments by ~9.5%, 14.87%, 13.79% and 10.43%, respectively, after 2, 4, 6 and 8 h of treatment. However, addition of NO to 250 μM Pb-treatment reduced MDA levels by ~ 18% after 2 h, whereas only 4.4% reduction was noticed after 8 h (Fig 1a).

Exposure to 50 and 250 μM Pb for 2−8 h enhanced CDs content by ~10–28% and ~23–60% over that of the control (Fig 1b). However, on SNP supplementation, CD content increased only by ~5−24% in 50 μM Pb+SNP treatment (significant at *p*<0.05) compared to control, when exposed for 2−8 h (Fig 1b). The extent of increase in CDs was lesser with SNP supplementation, thereby indicating its ameliorating potential in Pb exposed seedlings.

### Effect of NO on ROS Generation

**Fig 2:**
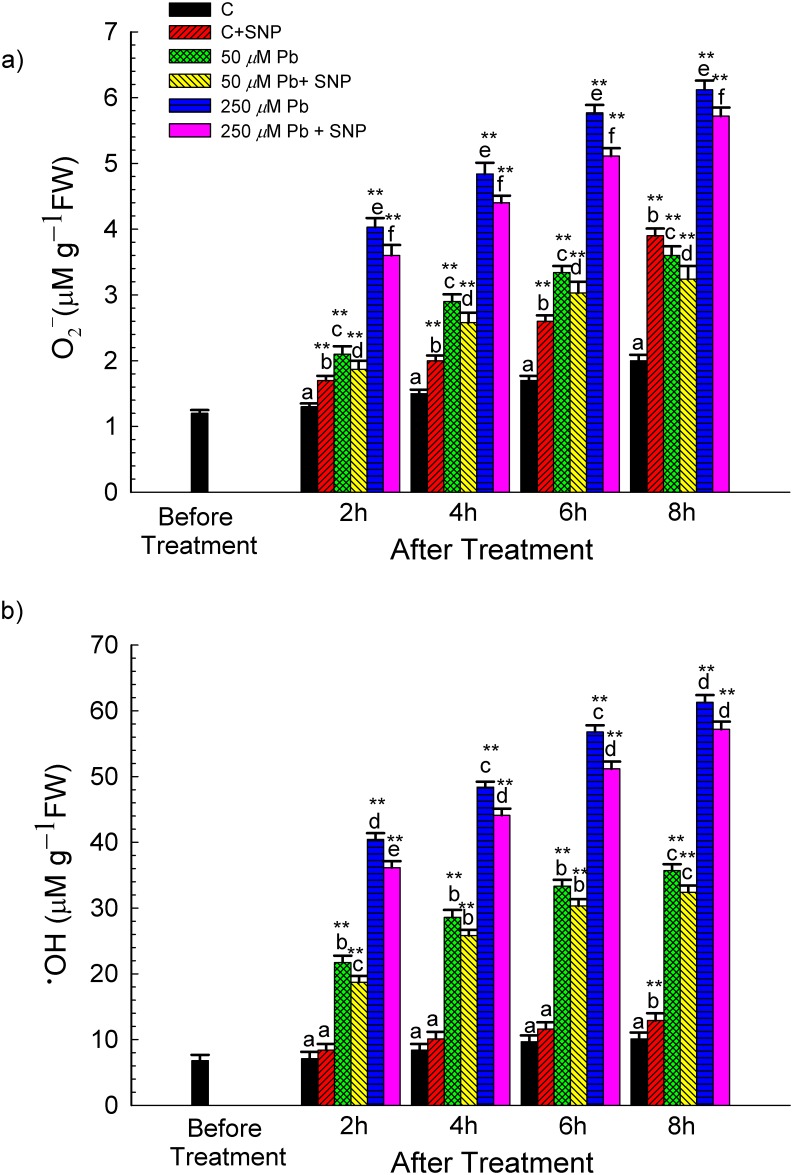
Effect of SNP (100 μM) on the Pb-induced changes in the content of (a) superoxide anion (O_2_^●−^, μM g^–1^ FW), and (b) hydroxyl ion (^●^HO, nM g^–1^ FW) of wheat seedlings determined before and after 2, 4, 6 and 8 h of exposure to Pb (50 and 250 μM, as lead nitrate). Data presented as mean±SE. * and ** represent significant difference of various treatments at 2, 4, 6 and 8 h stage from 0 h stage at *p*<0.05 and at *p*<0.01, respectively, applying post hoc Dunnett’s test. Different letters within a particular stage (2, 4, 6 or 8 h) represent significant difference at *p*<0.05 applying post hoc Tukey’s test.

O_2_^●−^ content increased by ~ 80% and 206% over the untreated control in response to 50 and 250 μM Pb, respectively, after 8 h of treatment (Fig 2a). However, SNP supplementation reduced O_2_^●−^ content by ~10% and 6.5% at 8 h compared to those treated with 50 μM and 250 μM Pb, respectively (Fig 2a).

**Fig 3:**
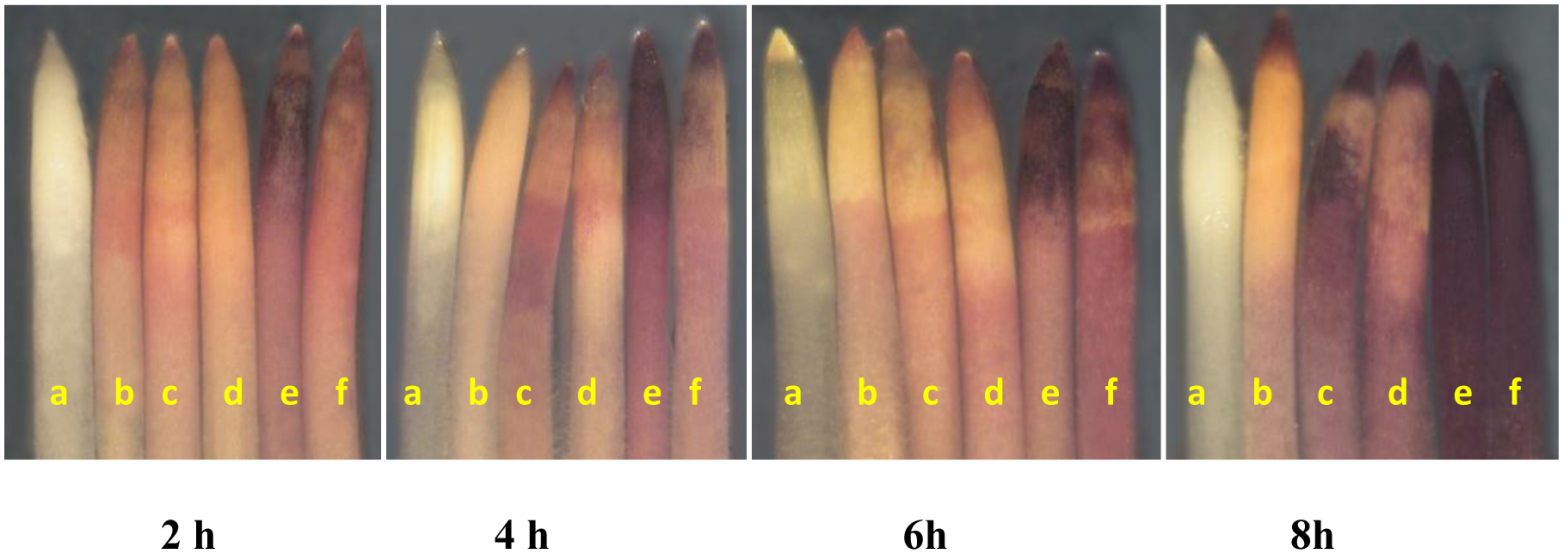
*In situ* histochemical localization showing the effect of SNP (100 μM) on superoxide ion (O_2_^●−^) generation in wheat roots after 2, 4, 6 and 8 h of Pb treatment. At each time period, roots from left to right inlcude: control (a), 100 μM SNP (b),50 μM Pb (c), 50 μM Pb+SNP (d), 250 μM Pb (e) and 250 μM Pb+SNP (f).

^**●**^HO content was enhanced by 205–253% and 470–507% during 2–8 h of exposure to 50 and 250 μM Pb, respectively, over that in the control. However, SNP reduced ^**●**^HO generation after 8 h by 10% and 7% in 50 and 250 μM Pb+SNP treatment, respectively, over Pb-alone treatments (Fig 2b). Likewise, H_2_O_2_ content increased by ~2.73–4.87 times and 3.73–6.06 times at 50 and 250 μM Pb-exposure, respectively, over that in the control, during 2–8 h of treatment (Fig 4a). Further, SNP supplementation ameliorated Pb (250 μM)-induced H_2_O_2_ accumulation by ~21.43% and 3.29% after 2 and 8 h of exposure, respectively as compared to Pb alone.

The quantitative changes in O_2_^●−^ levels were confirmed by *in-situ* histochemical localization in wheat roots on Pb-alone or Pb+SNP exposure. Compared to only Pb-treated roots, roots from Pb+SNP treatments stained less, indicating reduced O_2_^●−^ content (Fig 3).

**Fig 4:**
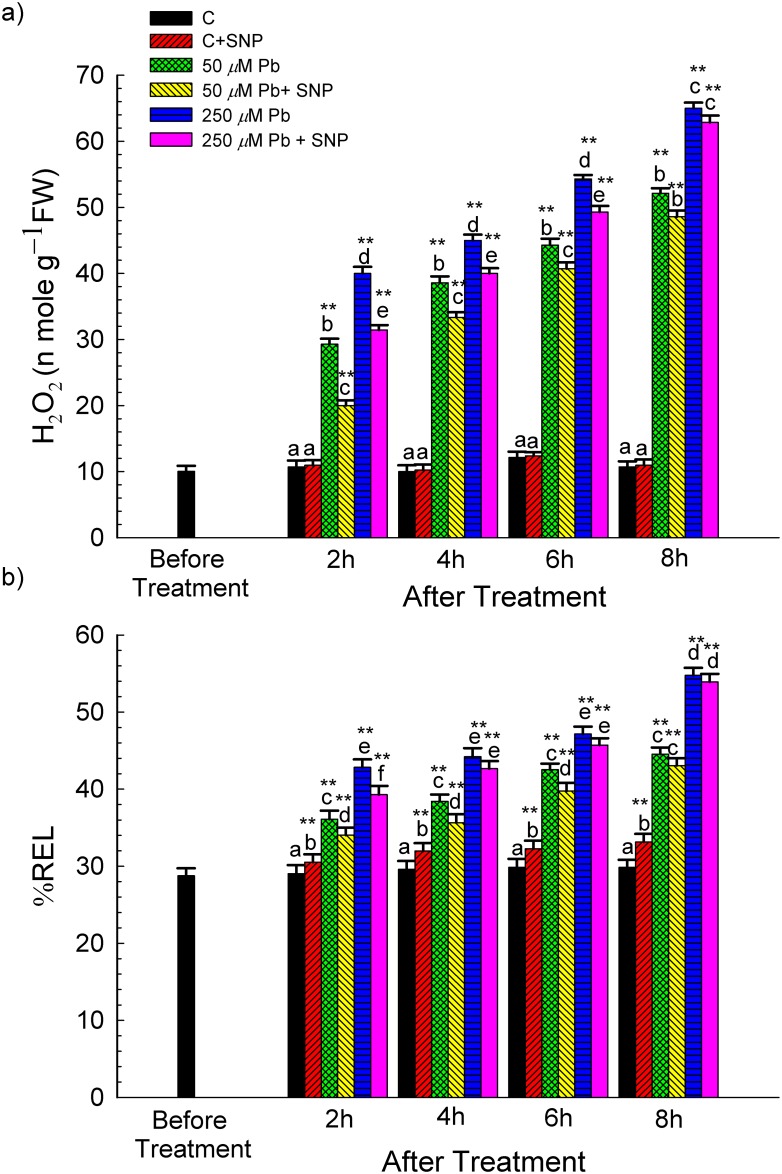
Effect of SNP (100 μM) on the Pb-induced changes in the (a) concentration of hydrogen peroxide (H_2_O_2_, nM g^–1^ FW) and (b) relative electrolyte leakage (REL, %) of wheat seedlings determined before and after 2, 4, 6 and 8 h of exposure to Pb (50 and 250 μM, as lead nitrate). Data presented as mean±SE. * and ** represent significant difference of various treatments at 2, 4, 6 and 8 h stage from 0 h stage at *p*<0.05 and at *p*<0.01, respectively, applying post hoc Dunnett’s test. Different letters within a particular stage (2, 4, 6 or 8 h) represent significant difference at *p*<0.05 applying post hoc Tukey’s test.

### Membrane Permeability

**Fig 5:**
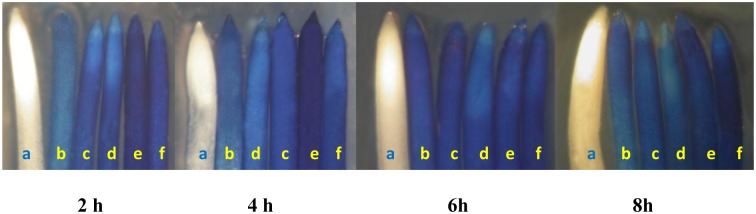
*In situ* histochemical localization showing the effect of SNP (100 μM) on Pb-induced loss of membrane integrity in wheat roots, after 2, 4, 6 and 8 h of Pb treatment. At each time period, roots inlcude: control (a), 100 μM SNP (b),50 μM Pb (c), 50 μM Pb+SNP (d), 250 μM Pb (e) and 250 μM Pb+SNP (f).

REL increased by ~24–49% and 48–83% at 50 and 250 μM Pb-exposure, respectively, over that in the control, from 2–8 h (Fig 4b). SNP supplementation significantly ameliorated Pb-toxicity during initial hours, i.e. up to 6 h in 50 μM Pb and up to 2 h in 250 μM Pb-treatment (Fig 4b). The ameliorating effect of SNP on membrane damage was further confirmed by *in vivo* staining of roots with Evans blue. Roots from Pb+SNP treatments stained less than those from Pb-alone treatments indicating an ameliorating / protective effect of SNP (Fig 5).

### Effect on NO Levels

**Fig 6:**
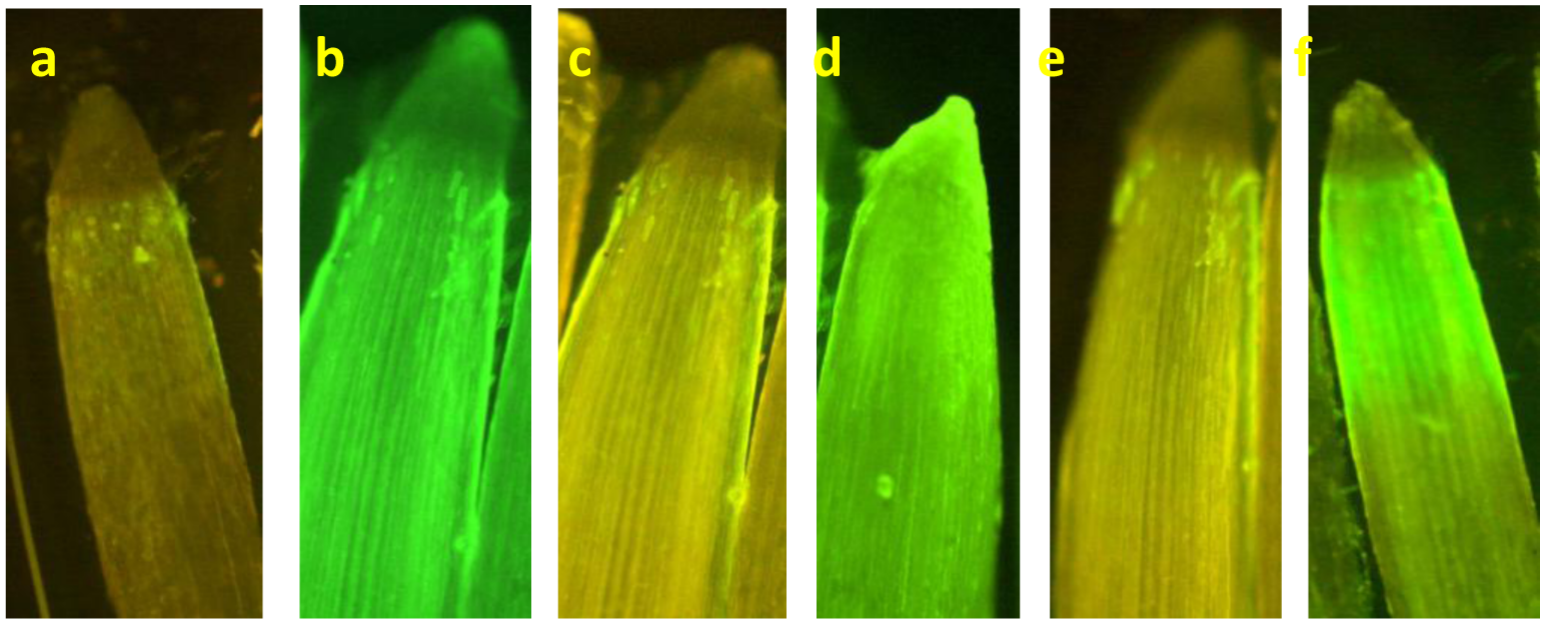
NO production in wheat roots after 8 h of treatment with Pb and SNP. Root samples were stained for NO with DAF-2DA and were investigated under fluorescence microscope. (a) Control, (b) 100 μM SNP, (c) 50 μM Pb, (d) 50 μM Pb+ SNP, (e) 250 μM Pb, and (f) 250 μM Pb+ SNP.

To explore whether Pb-induced oxidative damage was associated with any alteration in the level of NO, intracellular nitrite (NO_2_^−^) content was measured. It was observed that compared to control, Pb-exposure significantly (*p*<0.05) decreased NO level in wheat roots in a concentration-dependent manner over 2–8 h of exposure period (Table 2). After 4 h, NO levels decreased by nearly 4% and 33% at 50 and 250 μM Pb exposure, respectively, over the control. The intracellular nitrite content declined further and ~15% and 44% decrease was noticed after 8 h of exposure to 50 and 250 μM Pb, respectively, as compared to the control. However, after SNP supplementation, there was a significant increase in intracellular nitrite content in all the treatments including control (Table 2). Enhanced NO generation upon SNP supplementation was confirmed by *in situ* NO detection using DAF-2DA dye, wherein roots from Pb+SNP treatments exhibited greater fluorescence (Fig 6).

**Table 2:** Effect of 100 μM SNP on NO (μMg^−1^FW) generation in roots of wheat seedlings measured before treatment (referred as 0 h stage), and 2, 4, 6 and 8 h after treatment with Pb (50 and 250 μM, as lead nitrate).

Pb (μM)	Time after exposure (h)								
	0 h	2 h		4 h		6 h		8 h	
		−SNP	+SNP	−SNP	+SNP	−SNP	+SNP	−SNP	+SNP
0	97.1±0.35	98.2±0.02c	106.3±0.15a	94.1±0.15b	102.3±0.24a	106.3±0.54b	118.3±0.66b	170.0±0.45c	205.9±0.56f
50	−	94.1±0.19b	110.1±0.22b*	98.2±0.21c	114.2±0.43b*	103.3±0.45b	110.1±0.56a**	145.0±0.67b	172.0±0.71e*
250	−	66.2±0.17a	114.2±0.27c*	70.3±0.36a	118.3±0.41c*	78.2±0.38a	126.1±0.74c*	95.1±0.87a	122.1±0.79a*

Data represented as mean±SE of five independent replicates. Different letters in a column within a particular stage (2, 4, 6 or 8 h) represent significant difference among them at *p*<0.05 applying post hoc Tukey’s test.

* and ** represent significant difference between treatments (with and without SNP) at *p*<0.05 and at *p*<0.01, respectively, applying two-sample *t-*test

### Effect of NO on Scavenging Enzymes

**Fig 7:**
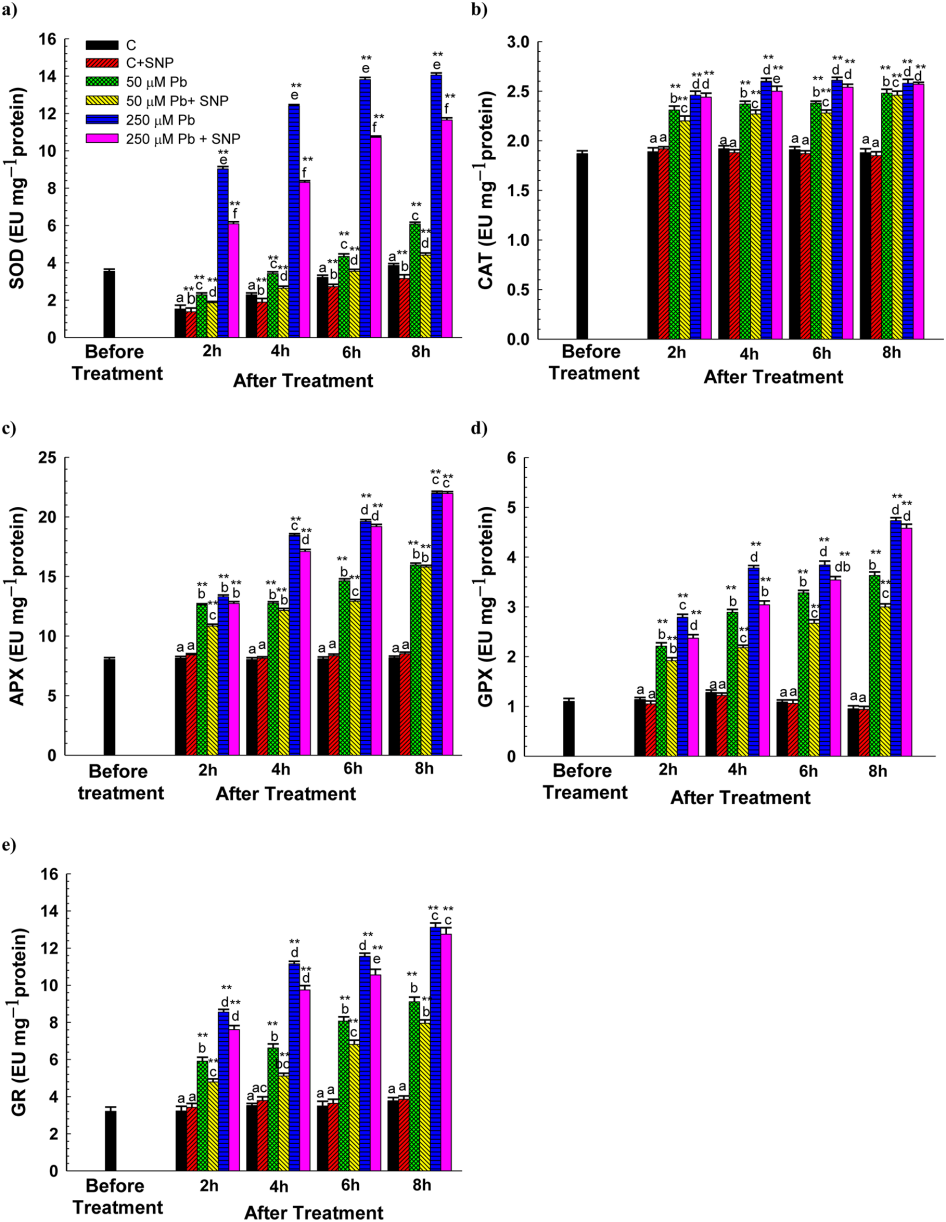
Effect of SNP (100 μM) on the Pb-induced changes in the specific activity of (a) SOD (EU mg^–1^ protein), (b) CAT (EU mg^–1^ protein), (c) APX (EU mg^–1^ protein), (d) GPX (EU mg^–1^ protein) and (e) GR (EU mg^-1^ protein) in the roots of wheat seedlings determined before treatment and after 2, 4, 6 and 8 h of exposure to Pb (50 and 250 μM, as lead nitrate). Data presented as mean±SE. * and ** represent significant difference of various treatments at 2, 4, 6 and 8 h stage from 0 h stage at *p*<0.05 and at *p*<0.01, respectively, applying post hoc Dunnett’s test. Different letters within a particular stage (2, 4, 6 or 8 h) represent significant difference at *p*<0.05 applying post hoc Tukey’s test.

Activities of scavenging enzymes (SOD, CAT, APX, GPX and GR) increased significantly in wheat roots on Pb-exposure in a dose- and time-dependent manner (Fig 7a–7e). The total soluble protein content was found to increase upon Pb exposure and decrease when supplemented with SNP. Gel electrograph indicates variations in the banding of protein patterns in root extracts of wheat in response to Pb and Pb+SNP treatments for 8 h (Fig 8.1). Exposure to 50 μM Pb enhanced SOD activity by 1.5–1.6- fold over a period of 2–8 h in comparison to their respective controls. However, SOD activity was higher than their respective control by 264% after 8 h of 250 μM Pb-treatment (Fig 7a). The quantitative changes were in accordance with findings of native SDS-PAGE gels. Distinctly resolved bands of SOD were clearly observed in the gel; however, the SOD activity was highest in the root extracts of wheat treated with 250 μM Pb after 8 h (Fig 8.2). CAT activity increased in the range of 22–32% and 30–37% upon exposure to 50 μM and 250 μM Pb, respectively, as compared to the control, over a period of 2–8 h (Fig 7b). The banding pattern of the CAT activity in the root extracts of wheat treated for 8 h clearly indicated that the Pb-treated roots synthesized more CAT than the control, and SNP was not able to ameliorate Pb-induced CAT activity (Fig 8.3). The activity of APX increased by ~1.5-, 1.6-, 1.8- and 2.0-times over that in the control after 2, 4, 6 and 8 h of exposure to 50 μM Pb (Fig 7c). Exposure to 250 μM Pb enhanced GPX activity by ~2.4-, 3.0-, 3.5- and 4.0-fold over the control after 2, 4, 6 and 8 h of exposure (Fig 7d). GR activity increased in the range of ~83–141% and 165–247% over 2–8 h of exposure to 50 and 250 μM Pb, respectively (Fig 7e). However, the induction levels of these scavenging enzymes (except CAT) were reduced upon SNP supplementation. SNP was able to successfully modulate Pb-induced SOD activity at both the concentrations of Pb (50 μM and 250 μM). The ameliorating effect of SNP on Pb-induced APX, GPX and GR upregulation was greater at 50 μM Pb than at 250 μM Pb treatments.

**Fig 8:**
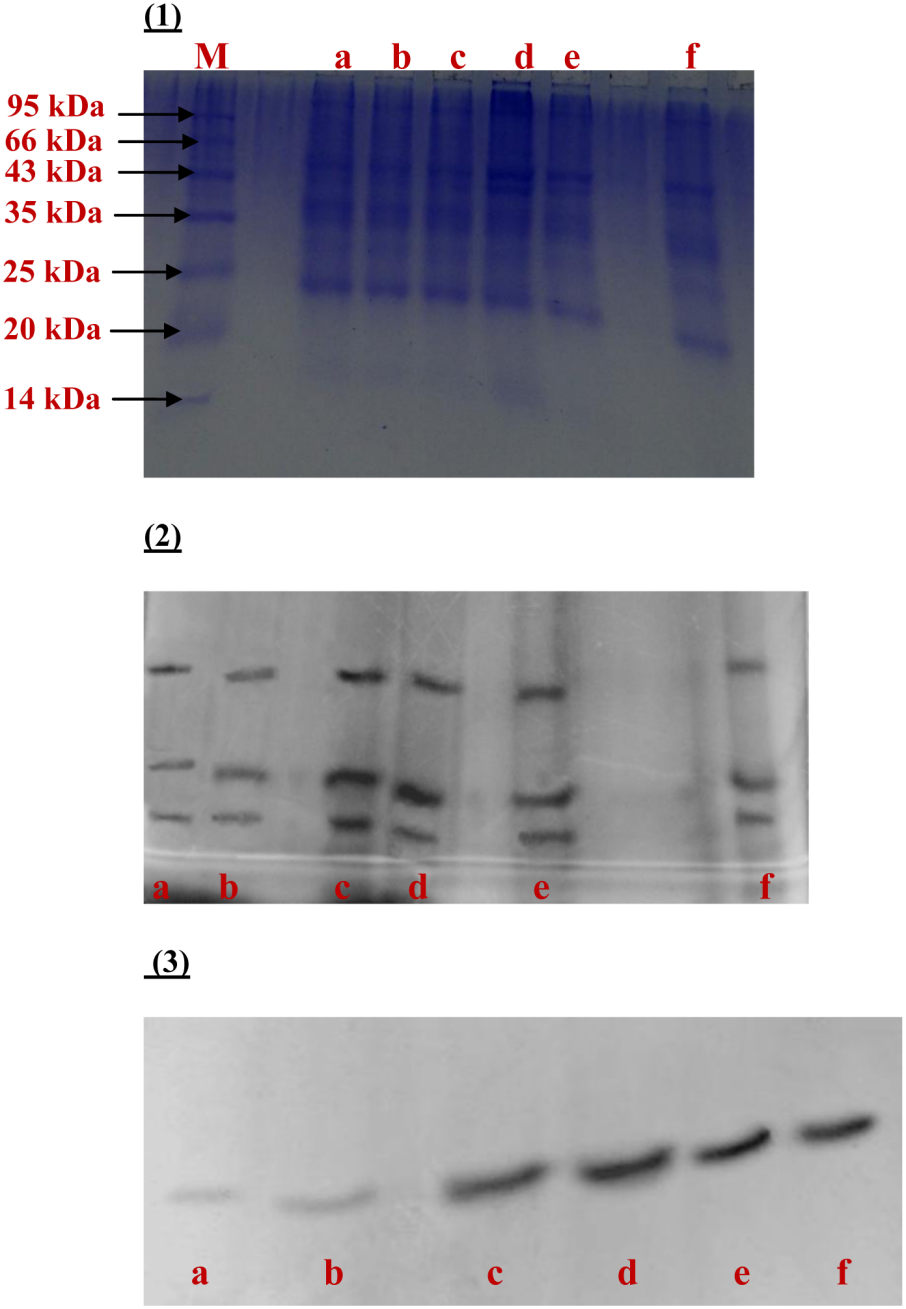
SDS–PAGE analysis of (1) The protein profile, (2) SOD activity, and (3) CAT activity in the root extracts of wheat treated for 8 h. In all the figures, a: control; b: 100 μM SNP; c: 50 μM Pb; d: 250 μM Pb; e: 250 μM Pb +SNP; f: 50 μM Pb +SNP.

## Discussion

Pb exposure significantly inhibited root growth and enhanced % REL and the contents of O_2_^●−^, H^●^HO, CDs, MDA and H_2_O_2_. SNP (a NO donor) addition alleviated Pb-toxicity as evidenced by improved radicle and plumule lengths; and decreased content of oxidative stress markers. However, this reduction was noticed only up to 6 h of Pb-treatment. Decreased ROS generation upon exogenous NO supplementation indicated ROS scavenging action of NO, and protection against Pb toxicity. These observations are corroborated by previous findings about the protective effect of NO against Cu [35], Al [36], Cd [22], and As [29]. A similar protective effect of SNP on H_2_O_2_-induced oxidative damage and senescence has been reported in rice leaves [37]. Nevertheless, exogenous NO has been reported to promote seed germination in lupin under Cd, Pb and salinity stress [38].

The findings that NO caused reduction in H_2_O_2_ accumulation in wheat roots due to Pb-toxicity is paralleled by earlier studies that NO inhibited H_2_O_2_ accumulation induced by water stress in maize [18], Cd in rice leaves [39], and UV-B in bean leaves [40]. Pb-exposure enhanced REL from wheat roots suggesting increased permeability due to membrane disruption. REL is an indicator of membrane damage and occurs due to membrane peroxidation resulting from an oxidative burst [41]. This membrane disruption is caused due to peroxidation of polyunsaturated fatty acids or lipids in the biomembranes to other byproducts (MDA and CDs) [42]. However, NO addition partially recuperated Pb-caused REL increase, suggesting a protective effect on membranes. These findings are supported by the earlier study of Shi *et al*. [40] who found that NO reduced the UV-B enhanced ion leakage in bean leaves. In our study, Pb-exposure increased the contents of TBARS and CDs; however, NO addition reduced their concentration. This reduction in the content of TBARS and CDs can be attributed to the ability of NO to directly scavenge ROS by acting as an antioxidant, and preventing accumulation of TBARS and CDs [43]. NO has been reported to be a potent inhibitor of lipid peroxidation [44]. NO exhibits both antioxidant and pro-oxidant activity that further depends on its application, location, and time of production in the cell [26]. Being an antioxidant, NO directly quenches ROS, including O_2_^●−^, and regulates oxidative damage. This is in agreement with the results obtained in our study where SNP addition paralleled a decrease in O_2_^●−^. In addition, NO has been documented to directly quench O_2_^●−^ and protect against oxidative stress induced by Fe-deficiency [45] and Cd and Pb exposure [38].

In the present study, Pb-exposure significantly decreased *in vivo* NO level and it correlated positively with ROS generation and oxidative damage. It parallels a similar report that As-treatment significantly reduces *in vivo* NO level in rice roots and induces oxidative damage [29]. However, exogenous NO (as SNP) supply significantly improves *in situ* NO levels in wheat roots and it corroborates decreased oxidative stress.

Pb-exposure enhanced the activities of scavenging enzymes−SOD, CAT, APX, GPX, and GR−in wheat roots. However, NO supply reduced the induction levels of these scavenging enzymes (except CAT), thereby suggesting direct involvement of NO in ameliorating Pb-induced oxidative stress. NO reduced Al-caused increase in the activity of scavenging enzymes in *Cassia tora* roots by 24−36% [36]. NO supplementation declined the activities of SOD, GPX, APX and GR that were enhanced under Cd treatment in hydroponically grown wheat roots [22]. In our study, the greatest effect of NO was on regulating the SOD activity. SODs have been regarded as the first line of defense against oxidative stress [46]. Previously, NO has been reported to partially prevent increase in the activity of SOD in sunflower leaves against Cd [43], in *Cassia tora* roots against Al [36], in wheat roots against Cd [22], and in rice roots against As [29]. The observed decrease in SOD induction on NO supply paralleled the reduction in O_2_^●−^ concentration quantified in NO supplemented treatments. However, this is in sharp contrast to an earlier finding that NO protects lupin roots against Pb-stress by enhancing SOD activity [38]. In wheat seedlings, exogenous SNP enhanced activities of SOD, APX and protein content, whereas decreased H_2_O_2_ and malondialdehyde content under Al stress [47]. The observed lower capacity of NO to decrease GR induction is supported by an earlier finding of Laspina *et al*. [43] who reported that NO did not modify Cd-induced changes in the GR activity in sunflower leaves. On the contrary, another study reported a significant reduction in GR activity upon NO supplementation in rice under As-stress [29].

In the current experiment, *in situ* NO levels increased significantly in wheat roots upon exogenous supply of NO with a simultaneous decrease in the oxidative stress caused by Pb toxicity. This quantitative estimation was also confirmed by *in situ* staining with NO sensitive dye DAF-2DA. It was used to monitor relative intracellular NO content using fluorescence microscopy. DAF-2DA fluorescence clearly indicates changes in NO within plant cells with a limitation to correspond changes in NO fluorescence with actual concentration of NO in solution. The present observations are in good agreement with the previous findings of Singh *et al*. [29] where exogenous NO improved *in situ* NO levels in rice roots treated with As. Rodríguez-Serrano [48] also observed reduction of *in vivo* NO levels in pea roots upon Cd exposure. Such an effect could be due to decline in the *NOS* (Nitric Oxide Synthase) activity following Pb exposure. Reduced *in situ* NO levels under Pb exposure suggest the protective role of exogenous NO against Pb-induced oxidative stress. However, we did not determine the activity of *NOS*, which could be elucidated in this regard.

In summary, the present study concludes that exogenous NO supply partially ameliorates Pb-toxicity and provide protection, but could not restore the plant growth on prolonged Pb-exposure. NO helped to ameliorate the oxidative stress, as it did not reverse the increased activity of the enzymes in response to increasing dose and duration of Pb-treatment. SNP ameliorated Pb-toxicity significantly during 0–6 h, which corresponds to its half life (i.e., the time period at which maximum concentration of NO from SNP solution was recorded). Nevertheless, further studies are required to elucidate the changes at the molecular levels and the particular isoenzymes/proteins that are involved in mediating the ameliorating action of NO.
